# Clinical and epidemiological characteristics of 646 hospitalised SARS-Cov-2 positive patients in Rivers State Nigeria: a prospective observational study

**DOI:** 10.11604/pamj.2021.38.25.26755

**Published:** 2021-01-12

**Authors:** Datonye Alasia, Golden Owhonda, Omosivie Maduka, Ifeoma Nwadiuto, Godswill Arugu, Charles Tobin-West, Esther Azi, Victor Oris-Onyiri, Inwon Joseph Urang, Victor Abikor, Ayo-Maria Olofinuka, Obelebra Adebiyi, Abiye Somiari, Hope Avundaa, Aloni Alali

**Affiliations:** 1 Department of Internal Medicine, University of Port Harcourt, Rivers State, Nigeria,; 2 Department of Public Health and Disease Control, Rivers State Ministry of Health, Rivers State, Nigeria,; 3 Department of Preventive and Social Medicine, University of Port Harcourt, Rivers State, Nigeria,; 4 Department of Community Medicine, Rivers State University Port-Harcourt, Nigeria, Rivers State, Nigeria,; 5 Department of Community Health, Rivers State Primary Health Care Management Board, Rivers State, Nigeria,; 6 World Health Organization Abuja Office, Abuja, Nigeria,; 7 Department of Community Medicine, University of Port Harcourt Teaching Hospital, Rivers State, Nigeria

**Keywords:** Epidemiology, characteristics, SARS-COV-2, COVID-19, Nigeria

## Abstract

**Introduction:**

the knowledge of epidemiologic and clinical variables in patients with SARS- CoV-2 infection provides evidence and lessons that are useful for the pandemic response, with consideration of National and sub-National variations. The objective of this study was to characterize and describe the clinical and epidemiologic features of all the hospitalised patients with COVID-19 in Rivers State Nigeria, from March to August 2020.

**Methods:**

a prospective descriptive multi-center study of patients with positive SARS-CoV-2 RT PCR, who were hospitalised for treatment and self-isolation in four treatment centers in Rivers state, Nigeria.

**Results:**

the mean age of all the patients was 39.21 ± 12.31 years, with a range of 2 to 77 years. The majority of patients were in the 31 to 40-year (33.0%), 41 to 50-year (23.1%) and 18-to 30-year (22.0%) age groups. The patient population included 474 (73.4%) males and 172 (26.6%) females, with 93 (14.4%) healthcare workers. A history of contact and travel was established in 38.5% and at least one comorbid disease condition was present in 32.8% of patients. Patients with severe disease were 61 (9.45%), while the overall case fatality rate was 2%. The leading comorbid disease conditions were Hypertension in 23.8% and diabetes in 7.7% of patients. Fever (26.0%), dry Cough (17.6%), dyspnoea (12.7%), anosmia (12.7%) and headache (9.9%) were the most common symptoms. The presence of comorbidity and increasing age predicted death from COVID-19.

**Conclusion:**

the clinical and epidemiologic characteristics of this cohort of hospitalised patients show significant similarities with existing trends from previously reported studies, with contextual peculiarities.

## Introduction

The COVID-19 pandemic which continues across the world has been associated with evolving clinical and epidemiologic features [1,2]. Initial and follow-up epidemiologic studies showed that majority (80%-81%) of patients diagnosed with COVID-19 usually have asymptomatic to mild disease, with the disease more prevalent in males and less common in children [2-4]. In the progression of the pandemic, similarities and variations have emerged in the clinical and epidemiologic trends of COVID-19 between and within continents, nations and regions [3,5]; indicating that demographic factors and local peculiarities influence the trends of the pandemic. The knowledge of these epidemiologic and clinical variables is therefore useful in understanding the pandemic and providing evidence and lessons to shape the response [6]. These epidemiological variations in COVID-19 are highlighted in a metanalysis by Ahmed *et al*. [5] comparing studies from eight countries, including, China, Italy, Australia, Canada, Korea, Taiwan, Singapore and the USA. The outcome indicates variations in symptom patterns and fatality rate amongst other findings. Further instances of the epidemiologic similarities and variation are demonstrated by a general epidemiologic study from Brazil, which revealed differences in epidemiologic trends characterised by the basic reproduction number (R0), among different regions in Brazil [7]. A hospital-based observational study from Spain [8] revealed higher admission rate in Latin Americans, while a study from south of Iran [9] showed more prevalence of atypical symptoms in hospitalised patients compared to European patterns with differences in fatality rate, in comparison with rates within and out of the country. Similarly, overall mortality rates were shown to be lower in a cohort of Chinese, hospitalised patients in Jingzhou compared to Wuhan [10].

A UNICEF report [2] indicates that there is a higher proportion of COVID-19 in people under 20 years of age in low-and middle-income countries (LMICs) in comparison to higher-income countries. It is therefore evident that the clinical and epidemiologic patterns of COVID-19 which vary across geographic areas are influenced by socio-economic and demographic variables [2,11,12] resulting in similar or differing trends in age and gender distribution, the spectrum of disease severity, symptomology, case fatality, rate of health worker infections and requirements for critical care amongst others. These descriptions of local clinical and epidemiological trends are relevant as they contribute to the local and global epidemiologic database of the disease which provides lessons for sustained public health response. From the time when Nigeria recorded her first case of COVID-19 in February 2020; the number of cases had continued to rise with Rivers state having the 7^th^ largest number of cases with 2180 reported cases as of 21^st^ June 2020 [13]. At the onset of the pandemic, the response in Rivers state and most of Nigeria was the hospitalisation, isolation and treatment of all positive cases of SAR-CoV-2 as a means of preventing onward transmission within the community. This model progressed until a combined model of home and hospital-based care was introduced in July 2020. The objective of this study was to characterize and describe the clinical and epidemiologic features of all the hospitalised patients with COVID-19 in Rivers state Nigeria, from March to August 2020.

## Methods

**Study sites and design:** this prospective study was performed in four (4) designated COVID-19 treatment centres in Rivers State (one of Nigeria´s 36 states with its capital in Port Harcourt). These treatment centres (study sites) included: Eleme Treatment Centre, Yakubu Gowon (CA-COVID) Treatment Centre, UPTH Treatment Centre, and the Bonny Isolation Centre.

**Study population, eligibility and data collection:** all consenting consecutive patients with a positive SARS-CoV-2 RT-PCR, who were hospitalised for treatment and self-isolation from the 25^th^ of March, 2020 to the 31^st^ of August, 2020 were recruited. A data extraction form built on the open data kit (ODK) tool to collect data from patients on admission, during hospitalisation, and on discharge was utilized. ODK was subsequently exported to a Microsoft Excel spreadsheet. Data domains included socio-demography, epidemiology, symptomatology, comorbidity and risk factor assessment, clinical variables, and treatment outcomes. Severity was classified using Nigerian Centre for disease control National COVID-19 case management guideline parameters [14] as severe and non-severe, with severity defined presence of fever > 38°C or suspected respiratory infection, plus one of respiratory rate > 30 breaths/min; severe respiratory distress; > SpO_2_ ≤ 93% on room air & Presence of co-morbid conditions such as diabetes, asthma, hypertension in adults and cough or difficulty in breathing & at least one of the following central cyanosis or SpO_2_ <92%; severe respiratory distress e.g. grunting breathing, very severe chest in-drawing & signs of pneumonia in children. Disease outcome was classified into discharged and died.

**Ethical considerations:** ethical approval was obtained from the Research Ethics Committee of the Rivers State Ministry of Health before the commencement of the study. Confidentiality was maintained by the removal of patient identifiers from the dataset and ensuring that only researchers involved in this study had access to the extracted data.

**Data analysis:** the data was exported from the Microsoft Excel spreadsheet into IBM Statistical Package for Social Sciences (SPSS) version 23 for the data analysis. Both descriptive and inferential analysis was conducted. Descriptive statistics through frequency tables were used in establishing the percentage of occurrence of various categorical study variables Continuous variables such as age and other biological and clinical parameters were compared for differences in their means using the independent student´s T-test. Pearson´s chi-square was used to test for significant differences between the values of categorical variables among persons with the severe and non-severe disease. Relative risk with 95% confidence intervals was used to determine the association between socio-demographic, epidemiological, and clinical characteristics as the independent variables and disease outcome as the dependent variables. A two-step logistic regression model was utilized to determine the predictors of disease outcome (alive or dead). Step one involved a bivariate logistic regression with crude odds ratio and 95% confidence intervals computed to determine associations between all socio-demographic (age, education, gender, and occupation) epidemiological and clinical characteristics (presenting symptoms and co-morbidity) as the independent variables and disease outcome as the dependent variable. The second step involved multivariable logistic regression with adjusted odds ratio and confidence intervals used to determine the predictors of disease outcome while controlling for known or unknown confounding variables. Variables that were included in the model include age, sex, occupation, and presence of co-morbidity. This was done to determine the predictors of dying from COVID-19. Output was presented as an adjusted odds ratio with p-values and 95% confidence intervals. For all associations, a p-value less than 0.05 was taken as significant.

## Results

**Socio-demographics of the study population:** a total of 646 hospitalised patients were recruited over the period. The mean age of all patients was 39.21 ± 12.31 years, with a range of 2 to 77 years. The majority of patients were in the 31-40 year (33.0%), 41-50 year (23.1%), and 18-30 year (22.0%) age groups, as shown in (Table 1). The patient population included 474 (73.4%) males and 172 (26.6%), females, while 553(85.6%) were non-healthcare workers and 93 (14.4%) were healthcare workers (Table 1).

**Table 1:** socio-demographic, epidemiological, clinical and outcome characteristics of patients

Variable	Frequency	Percentage
**Age group**		
<18	17	2.6
18-30	142	22.0
21-40	213	33.0
41-50	156	24.1
51-60	84	13.0
61-70	27	4.2
>70	7	1.1
**Gender (n=646)**		
Male	474	73.4
Female	172	26.6
**Occupation (n=646)**		
Healthcare worker	553	85.6
Non-healthcare worker	93	14.4
**Education (n=421)**		
Primary	7	1.7
Secondary	60	14.3
Tertiary	354	84.1
**Source of contact (n=646)**		
Known	255	39.5
Unknown	391	60.5
**Comorbidity (n=646)**		
Present	212	32.8
Absent	434	67.2
**Disease Severity (n=646)**		
Non-Severe	585	90.6
Severe	61	9.4
**Disease Outcome (n=646)**		
Discharged	633	98.0
Died	13	2.0

**Epidemiologic, clinical, and outcome characteristics:** a history of contact was known in 255 (38.5%) of patients while the source of infection was unknown in 391 (60.5%) patients (Table 1). Patients with severe disease were 61 (9.45%) compared to 585 (90.6%) without the severe disease, while 633 (98.0%) were discharged and 13 patients died giving a case fatality rate (CFR) of 2% (Table 1).

**The pattern of comorbidities and symptoms of the disease:** the distribution of comorbid disease conditions and clinical symptoms in the patients is presented in Table 2. At least one comorbid disease was present in 212 (32.8%) of the study population compared to 434 (67.2%) without comorbidity (Table 1). The leading comorbid disease conditions were hypertension in 157 (23.8%) and Diabetes in 50 (7.7%) (Table 2). The leading symptoms at presentation were Fever 162 (26.0%), Dry cough 114 (17.6%), dyspnoea 82(12.7%), and anosmia 82(12.7%) (Table 2). Other symptoms of interest were intractable hiccups in 3 patients and acute confusion state/delirium in 2 patients.

**Table 2:** pattern of clinical symptoms and co-morbidities in patients

Variable	Frequency	Percentage (%)
**Presenting symptoms**		
Fever	168	26.0
Dry Cough	114	17.6
Dyspnea	81	12.5
Anosmia	75	11.6
Headache	64	9.9
Fatigue	59	9.1
Myalgia	55	8.5
Aguesia	41	6.3
Productive cough	34	5.3
Diarrhoea	27	4.2
Sore throat	24	3.7
Rhinorrhoea	21	3.3
Vomiting	12	1.9
Others	40	6.2
**Comorbidity**		
Hypertension	157	23.8
Diabetes	50	7.7
Asthma	7	1.1
Heart Disease	5	0.8
Kidney Disease	4	0.6
HIV/AIDS	2	0.3
COPD	1	0.2

**Comparison of Baseline clinical parameters by disease severity and outcome:** the comparison of baseline clinical parameters in patients with the severe and non-severe disease and discharged and dead patients are shown in Table 3. Respiratory rate per minute (34.11±10.58 vs.21.46±3.08), temperature (°C) (37.11±0.92 vs.36.53±0.41), and pulse rate per minute (107.08±15.39 vs.85.40±13.25), respectively, were significantly higher in patients with severe disease; while SPO_2_ (%) (80.93±17.27 vs .98.02±1.47) was significantly lower in patients with severe disease, (Table 3). Patients who died had significantly higher respiratory rate per minute (41.54±10.81 vs.22.64±5.42), pulse rate per minute (117.85±7.83 vs. 86.80±14.32), and systolic blood pressure (mmHg) (140.50±13.55 vs. 132.14±18.92). The SPO^2^ (%) was lower in patients who died (66.85±20.37 vs. 97.02±5.39) (Table 3).

**Table 3:** comparison of admission vital signs across disease severity and outcomes

	Mean ±SD		p-value	Mean ±SD		P-value
Vital signs on admission	Severe (n =61)	Non-severe (n = 585 )		Dead (n = 13)	Discharged (n = 633)	
SPO_2_ (%)	80.93±17.27	98.02±1.47	<0.001	66.85±20.37	97.02±5.39	<0.001
Respiratory rate (/minute)	34.11±10.58	21.46±3.077	<0.001	41.54±10.81	22.64±5.42	<0.001
Temp (°C)	37.11±0.92	36.53±0.41	<0.001	36.77±0.44	36.58±0.50	0.27
Pulse (beats/minute)	107.08±15.39	85.40±13.25	<0.001	117.85±7.83	86.80±14.32	<0.001
Systolic blood pressure (mmHg)	134.88±20.02	132.03±18.73	0.27	140.50±13.55	132.14±18.92	0.03
Diastolic blood pressure (mmHg)	82.84±14.13	85.04±12.71	0.22	89.75±17.55	84.75±12.74	0.18

**Comparison of mean age across socio-demographic and clinical parameters:** the comparison of mean age by socio-demographic and clinical parameters is shown in Table 4. Persons with severe disease were significantly older 52.49 (14.35) years vs 37.83 (11.22) years t=7.74; p=<0.001. Persons who died were also significantly older 54.92 (10.94) years vs 38.89 (12.13) years t=-4.73; p=<0.001, (Table 4).

**Table 4:** comparison of mean age across socio-demographic and clinical parameters

Variable	Mean (SD)	T-test	p-value
**Gender**			
Male	40.57 (11.84)	4.73	<0.001*
Female	35.47 (12.83)		
**Occupation**			
Non-healthcare worker	39.12 (12.48)	-0.47	0.64
Health care worker	39.76 (11.24)		
**Disease Severity**			
Severe	52.49 (14.35)	7.74	<0.001*
Non-severe	37.83 (11.22)		
**Outcome**			
Discharged	38.89 (12.13)	-4.73	<0.001*
Dead	54.92 (10.94)		

**Associations between socio-demographic variables, co-morbidity and disease severity and outcomes:** the effect of demographic and comorbid factors on disease severity and outcome is shown in Table 5. The significant variables which influenced disease severity and adverse outcomes (death) were the presence of age above 50 years and the presence of comorbidities. Patients aged 51 years and above, were significantly more likely to have the severe disease with (Adj O.R = 1.41, 95% C.I = 1.24 -1.60 and p <0.001) compared to patients below 50 years (Table 5). Patients aged 51 years and above, were significantly more likely to die with (R.R = 1.07, 95% C.1 = 1.02- 1.13, p <0.001), compared to patients below 50 years (Table 5). Patients with comorbidities were significantly more likely to have the severe disease (O.R = 10.44; 95% C.I = 5.41 - 20.15, p <0.001) compared to patients without comorbidities (Table 5). Patients with comorbidities were significantly more likely to die than those without comorbidity, (RR = 24.57; 95% C.I =3.22 to 187.68, p <0.001) (Table 5).

**Table 5:** associations between socio-demographic variables, co-morbidity and disease severity and outcome

Variable	Severe	Non-Severe	Chi-square (p-value)	Odds Ratio (95% C.I)
	N (%)	N (%)		
**Age category**				
50 years and below	23 (37.7)	505 (86.3)	87.46 (<0.001)	1.41 (1,24 -1.60)
51 years and above	38 (62.3)	80 (13.7)		
**Education**				
Primary and Secondary	7 (15.2)	60 (16.0)	0.02 (0.89)	1.01 (0.92 - 1.10)
Tertiary	39 (84.8)	315 (84.0)		
**Sex**				
Male	51 (83.6)	423 (72.3)	3.6 (0.06)	1.85 (0.96 - 3.56)
Female	10 (16.4)	162 (27.7)		
**Occupation**				
Non-healthcare worker	54 (88.5)	499 (85,3)	0.47 (0.50)	1.30 (0.61 - 2.76)
Health-care worker	7 (11.5)	86 (14.7)		
**Comorbidity**				
Yes	51 (83.6)	161 (27.5)	78.81 (<0.001)	10.44 (5.41 - 20.15)
No	10 (16.4)	424 (72.5)		
**Variable**	**Discharged**	**Died**	**Chi-square (p-value)**	**Relative Risk (95% C.I)**
**Age category**	N (%)	N (%)		
50 years and below	524 (82.8)	4 (30.8)	<0.001+*	1.07 (1.02-1.13)*
51 years and above	109 (17.2)	9 (69.2)		
**Education**				
Primary and Secondary	7 (15.2)	60 (16.0)	0.02 (0.89)	1.76 (0.94-1.03)
Tertiary	39 (84.8)	315 (84.0)		
**Sex**				
Male	462 (73.0)	12 (92.3)	0.20+	4.35 (0.57 - 33.24)
Female	171 (27.0)	1 (7.7)		
**Occupation**				
Non-healthcare worker	542 (85.6)	11 (84.6)	1.0+	0.93 (0.21-4.11)
Health-care worker	91 (14.4)	2 (15.4)		
**Comorbidity**				
Yes	200 (31.6)	12 (92.3)	<0.001+*	24.57 (3.22 -187.68)*
No	433 (68.4)	1 (7.7)		

+ Fisher's exact *significant association

**Associations between presenting symptoms, comorbidities and disease severity:** the proportional occurrence of severe disease based on symptoms and comorbidities is displayed in Table 6. Fever was significantly more frequent in patients with severe disease (73.8%) compared to those without severe disease (21%) with (O.R = 10.0, C.I = 5.77 - 19.33), p <0.001) (Table 6). Dry cough was significantly more frequent in patients with severe disease (42.6%) compared to those without severe disease (15.0%) (O.R = 4.20, C.I = 2.41 - 7.31) p <0.001), (Table 6). Dyspnea was significantly more frequent in patients with severe disease (73.3%) compared to those without severe disease (6.3%), with (O.R = 40.73, C.I = 21.01-78.96 5, p <0.001) (Table 6). Fatigue was significantly more frequent in patients with severe disease (34.4%) compared to those without severe disease (6.5%), with (O.R = 7.56, C.I = 4.06-14.08, p <0.001), (see, table 6). Productive cough was significantly more frequent in patients with severe disease (21.2%) compared to those without severe disease (3.6%), with (O.R = 7.27, C.I = 0.34-15.43, p <0.001), (see, table 6). Diarrhoea was significantly more frequent in patients with severe disease (16.4%) compared to those without severe disease (2.9%), with (O.R = 6.54, C.I = 2.85-15.03, p <0.001) (Table 6). Vomiting was significantly more frequent in patients with severe disease (6.6%) compared to those without severe disease (1.4%), with (O.R = 5.05, CI = 1.48-17.30, p = 0.004), (see, table 6). In patients with hypertension, severe disease was significantly more frequent (65.6%) compared to non-severe disease (19.5%), with (O.R = 7.87, C.I = 4.47-13.87, p <0.001) (Table 6). In patients with diabetes, severe disease was more significantly frequent in (31.1%) compared to non-severe disease (5.3%), with (O.R = 8.08, C.I = 4.21-15.51, p <0.001) (Table 6). In patients with kidney disease, the severe disease was more significantly frequent (6.6%) compared to non-severe disease (0.0%), with p <0.001 (Table 6). In patients with heart disease, severe disease was more significantly frequent (6.6%) compared to non-severe disease (0.2%), with (O.R = 40.98, C.I = 4.50-372.88, p <0.001) (Table 6).

**Table 6:** associations between symptoms, co-morbidities and disease severity

Variable	Severe	Non-Severe	Chi-square (p-value)	Odds Ratio (95% C.I)		Severe	Non-Severe	Chi-square (p-value)	Odds Ratio (95% C.I)
Presenting symptoms	N (%)	N (%)			Presenting Symptoms	N (%)	N (%)		
**Fever**					**Sore throat**				
Yes	45 (73.8)	123 (21.0)			Yes	3(4.9)	21(3.6)		
No	16 (26.2)	462 (79.0)	(<0.001)	10.0 (5.77 - 19.33)	No	58(95.1)	563(96.4)	(0.60)	1.39(0.40-4.79)
**Dry Cough**					**Rhinorhea**				
Yes	26 (42.6)	88 (15.0)			Yes	1(1.6)	20(3.4)		
No	35 (57.4)	497 (85.0)	(<0.001)	4.20 (2.41 - 7.31)	No	60(98.4)	564(96.6)	(0.46)	0.47(0.06-3.56)
**Dyspnea**					**Vomitting**				
Yes	44(73.3)	37(6.3)			Yes	4(6.6)	8(1.4)		
No	16(26.7)	548(93.7)	(<0.001)	40.73 (21.01-78.96)	No	57(93.4)	576(98.6)	(0.004)	5.05(1.48-17.30
					**Comorbidity**	N (%)	N (%)		
**Anosmia**					**Hypertension**				
Yes	6(10.0)	69(11.8)			Yes	40(65.6)	114(19.5)		
No	54(90.0)	514(88.2)	(0.67)	0.83 (0.34-2.00)	No	21(34.4)	471(80.5)	(<0.001)	7.87(4.47-13.87)
**Headache**					**Diabetes**				
Yes	5(8.2)	59(10.1)			Yes	19(31.1)	31(5.3)		
No	56(91.8)	526(89.9)	(0.64)	0.80 (0.31-2.07)	No	42(68.9)	554(94.7)	(<0.001)	8.08(4.21-15.51)
**Fatigue**					**Asthma**				
Yes	21(34.4)	38(6.5)			Yes	1(1.6)	6(1.0)		
No	40(65.6)	547(93.5)	(<0.001)	7.56 (4.06-14.08)	No	60(98.4)	579(99.0)	(0.66)	1.61(0.19-13.58)
**Myalgia**					**Heart Disease**				
Yes	9(14.8)	46(7.9)			Yes	4(6.6)	1(0.2)		
No	52(85.2)	536(92.1)	(0.07)	2.03 (0.94-4.38)	No	57(93.4)	584(99.8)	(<0.001)	40.98(4.50-372.88)
**Aguesia**					**Kidney Disease**				
Yes	3(4.9)	38(6.5)			Yes	4(6.6)	0(0.0)		
No	58(95.1)	547(93.5)	(0.63)	0.75 (0.22-2.49)	No	57(93.4)	585(100.0)	(<0.001)	NA
**Productive cough**					**HIV/AIDS**				
Yes	13(21.2)	21(3.6)			Yes	1(1.6)	1(0.2)		
No	48(78.7)	564(96.4)	(<0.001)	7.27 (0.34-15.43)	No	60(98.4)	584(99.8)	(0.04)	9.37(0.60-157.60)
**Diarhoea**					**COPD**				
Yes	10(16.4)	17(2.9)			Yes	1(1.6)	1(0.2)		
No	51(83.6)	567(97.1)	(<0.001)	6.54 (2.85-15.03)	No	60(98.4)	581(99.8)	(0.05)	9.68(0.60-156.79)

NA: Not applicable because O.R was not computed for variables with values of 0 in any cell

**Associations between presenting symptoms, comorbidities, and disease outcome:** the proportional occurrence of survival or death based on symptoms and comorbidities is displayed in Table 7. In patients with fever the proportion of those who died (61.5%) was significantly higher than those who were discharged (25.3%), with (R.R = 0.21, C.I = 0.07-0.66, p = 0.003), (Table 7). In patients with dry cough the proportion of those who died (53.8%) was significantly higher than those who were discharged (16.9%), with (R.R = 0.17, C.I = 0.06-0.53, p = 0.001), (see, Table 7). In patients with dyspnea the proportion of those who died (76.9%) was significantly higher than those who were discharged (11.2%), with (R.R = 0.04, C.I = 0.10-0.14, p <0.001) (Table 7). In patients with fatigue the proportion of those who died (38.5%) was significantly higher than those who were discharged (8.5%), with (R.R = 0.15, C.I = 0.05-0.47, p <0.001) (Table 7). In patient with productive cough the proportion of those who died (23.1%) was significantly higher than those who were discharged (4.9%), with (R.R = 0.17, C.I = 0.05-0.66, p = 0.004) (Table 7). A significantly higher proportion (69.2%) of patients with hypertension died compared to those who were discharged (22.9%), with (R.R = 0.13, C.I = 0.04-0.44, p <0.001) (Table 7). A significantly higher proportion (30.8%) of patients with diabetes died compared to those who were discharged (7.3%), with (R.R = 0.18, C.I = 0.05-0.60, p = 0.002), (Table 7). A significantly higher proportion (7.7%) of patients with heart disease died compared to those who were discharged (0.6%), with (R.R = 0.08, C.I = 0.01-0.73, p = 0.004) (Table 7).

**Table 7:** association between clinical symptoms, co-morbidities and treatment outcomes

Variable	Discharged	Died	Chi-square (p-value)	Relative Risk (95% C.I)	Variable	Discharged	Died	Chi-square (p-value)	Relative Risk (95% C.I)
Presenting symptoms	N (%)	N (%)			Presenting symptoms	N (%)	N (%)		
**Fever**					**Sore throat**				
Yes	160(25.3)	8(61.5)	(0.003)	0.21(0.07-0.66)	Yes	24(3.8)	0(0.0)	(0.474)	NA
No	473(74.7)	5(38.5)			No	608(96.2)	13(100.0)		
**Dry Cough**					**Rhinorhea**				
Yes	107(16.9)	7(53.8)	(0.001)	0.17(0.06-0.53)	Yes	21(3.3)	0(0.0)	(0.504)	NA
No	526(83.1)	6(46.2)			No	611(86.7)	13(100.0)		
**Dyspnea**					**Vomiting**				
Yes	71(11.2)	10(76.9)	(<0.001)	0.04(0.10-0.14)	Yes	12(1.9)	0(0.0)	(0.616)	NA
No	561(88.8)	3(23.1)			No	620(98.1)	13(100.0)		
					**Comorbidity**				
**Anosmia**					**Hypertension**				
Yes	75(11.9)	0(0.0)	(0.204)	NA	Yes	145(22.9)	9(69.2)	(<0.001)	0.13(0.04-0.44)
No	556(88.1)	12(100.0)			No	488(77.1)	4(30.8)		
**Headache**					**Diabetes**				
Yes	64(10.1)	0(0.0)	(0.227)	NA	Yes	46(7.3)	4(30.8)	(0.002)	0.18(0.05-0.60)
No	569(89.9)	13(100.0)			No	587(92.7)	9(69.2)		
**Fatigue**					**Asthma**				
Yes	54(8.5)	5(38.5)	(<0.001)	0.15(0.05-0.47)	Yes	7(1.1)	0(0.0)	(0.703)	NA
No	579(91.5)	8(61.5)			No	626(98.9)	13(100.0)		
**Myalgia**					**Heart Disease**				
Yes	54(8.5)	1(7.7)	(0.915)	1.12(0.14-8.77)	Yes	4(0.6)	1(7.7)	(0.004)	0.08(0.01-0.73)
No	579(91.5)	12(92.3)			No	629(99.4)	12(92.3)		
**Aguesia**					**Kidney Disease**				
Yes	41(6.5)	0(0.0)	(0.343)	NA	Yes	4(0.6)	0(0.0)	(0.774)	NA
No	592(93.5)	13(100.0)			No	629(99.4)	13(100.0)		
**Productive cough**					**HIV/AIDS**				
Yes	31(4.9)	3(23.1)	(0.004)	0.17(0.05-0.66)	Yes	2(0.3)	0(0.0)	(0.839)	NA
No	602(95.1)	10(76.9)			No	631(99.7)	13(100.0)		
**Diarhoea**					**COPD**				
Yes	26(4.1)	1(7.7)	(0.24)	0.52 (0.06-4.1)	Yes	2(0.3)	0(0.0)	(0.839)	NA
No	606(95.9)	12(92.3)			No	628(99.7)	13(100.0)		

NA: Not applicable because O.R was not computed for variables with values of 0 in any cell

**Predictors of death in patients:** with multivariable logistic regression analysis, the significant predictors of death were the presence of co-morbidities with (Adj O.R = 11.3; 95% CI = 1.34 to 95.76), p = 0.03 and increasing age > 50 years (Adj O.R = 1.07; 95% CI = 1.02-1.13), p-value = 0.01.

## Discussion

The knowledge of clinical and epidemiologic characteristics of SARS-CoV-2 infection provides information to guide the response to the pandemic. This prospective study describes the clinical and epidemiologic characteristics of hospitalised SARS-CoV-2 positive patients, in Rivers State, Nigeria. The age of patients with COVID-19 has been documented as a key determinant of disease presentation and clinical outcome, with population demographics significantly influencing these patterns [11,15]. The age pattern found in this study is close to the findings by Zamparini *et al*. [16], in hospitalised patients in South Africa with a median age of 42 years and the majority of the patients between the 18 to 50 year age groups. Bowale *et al*. [17] and Otounye *et al*. [18] in descriptive studies of hospitalised patients in Lagos, reported comparable mean ages with almost half of the patients below 40 years. Shahriarirad *et al*. [9], in Iran, established a higher mean age while De Souza *et al*. [7] reported a median age of 59 years in a Brazilian cohort, with the majority of the patients aged ≥50 years. In contrast to this study, Giesen *et al*. [8] in a study of hospitalised patients in Spain, found that the median age of patients was higher at 64.4 years, with only 4.5% of patients below 29 years and almost half above 70 years. The outcomes from this study validate previous findings from Nigeria and Africa [16-18]; showing younger COVID-19 patients in contrast to findings from Europe, North and South America [5,7,8]. Also the findings in this study validate a previous UNICEF report which indicates that there is a higher proportion of COVID-19 in people under 20 years of age in low and middle- income countries (LMICs) in comparison to higher-income countries.

Most patients in this study were males. This finding is consistent with other reports and the pattern across the globe which report more males than females though with varied proportions. Two studies [17,18] in Lagos Nigeria also reported the majority of hospitalised patients as males. The observations from other countries are similar to studies from Iran [9], China [19], Brazil [8], and Spain [7]. Healthcare worker infection has been a major source of concern due to its impact on the availability of already strained and inadequate frontline health human resources. In this study, 14.4% of the hospitalised patients were healthcare workers. This observed prevalence is high when compared to reports from Spain [7], Iran [9], and a Chinese cohort [3]. The observed higher numbers of healthcare workers may be a reflection of infection prevention and control (IPC) knowledge and practice as well as the availability of protective wear and appropriate health facility design. The perception of poor workplace safety and inadequacy in PPE availability has previously been reported in a study among healthcare workers in South-South, Nigeria [20]. This finding calls for the intensification of IPC knowledge and practice among healthcare workers.

The history of known contact and travel to areas of transmission were key indices in the initial case definition and epidemiologic response; while an inability to establish a contact and the source of infection indicates the occurrence of community transmission. In this study history of contact was established in just over one-third of patients while the source of infection was unknown in almost two-thirds of patients. Similar findings of lower percentage with a source of contact have been also been reported in Iran [9] and South Africa [16]. The presence of comorbidities that predispose to severe disease has been found to impact adversely on COVID-19 outcomes [19,21]. At least one comorbid disease condition was present in one-third of the patients in this study. This is lower than figures reported by Zhou *et al*. [19], Otuonye *et al*. [18], Zamparini *et al*. [16], and Shahriarirad *et al*. [9], respectively. The relatively lower proportion of patients with comorbidity in this study may be explained by the younger age of the participants in this study and the admission of asymptomatic patients for self-isolation. The analysis of the spectrum of comorbidities showed that hypertension and diabetes were the leading comorbidities in this study. These findings are consistent with that of other studies [16,18,19] that report hypertension and diabetes as the leading comorbidities in persons with COVID-19.

The symptoms associated with COVID-19 have evolved with documented geographic variations in the pattern and spectrum of symptoms [5]. The leading symptoms at presentation in this study were fever, dry cough^2^, dyspnoea, anosmia, and headaches. A similar trend has been observed in Nigeria by Bowale *et al*. [17]. Another study [18], in Lagos, reported malaise, dyspnoea, fever, dry cough, chest pain, anorexia, joint pain, diarrhoea, abdominal pain, sore throat, anosmia ,and ageusia, as the leading symptoms. Zamparini *et al*. [16], in South Africa also reported cough, fever, and Dyspnoea as the leading symptoms. A metanalysis [5] of 13 studies across 8 countries (China, Italy, Australia, Canada, Korea, Taiwan, Singapore, and the USA) still show that fever, cough, dyspnoea, sore throat, diarrhoea, nasal congestion, headache, sputum production, fatigue, myalgia remain the leading symptoms, with variations in the pattern of predominance across countries and continents. In this study, other symptoms of interest were intractable hiccups in three patients and acute confusion & delirium in two patients. The observation of hiccup as an atypical presentation of COVID-19 has earlier been reported by Prince and Sergel [21]. The findings of these symptoms (hiccups and delirium) show the evolving nature of symptoms associated with COVID-19 and buttresses the need for continued surveillance. The results of this study also show that fever, dry cough, dyspnoea, productive cough, and diarrhoea were more likely to occur in patients with severe disease. Disease severity patterns in this study are similar to the findings by Shahriarirad *et al*. [9] in Iran and Bowale *et al*. [17] in Lagos but lower than the prevalence found by Wu *et al*. [3] in China. The overall case fatality rate (CFR) in this hospitalised cohort was 2.0%. This is consistent with the finding from other parts of Nigeria [18] and China [3] respectively and far lower than observations by Giesen *et al*. [8] in Spain. These differences could be explained by the variations in age and prevalence of comorbidities in various populations. The impact and pattern of various demographic and clinical variables on disease severity and hospitalisation outcomes showed that patients with a comorbid disease had a significantly higher frequency of severe disease and death compared to those without comorbidity. The association of worsening disease severity and death found in this study also corresponds with previous observations [5,19] and highlights the importance of prevention and protection of persons with comorbidities from COVID-19. Hypertension and diabetes are the leading comorbidities that impact disease severity and outcomes [5,19,22]. The patients with hypertension in this study, had a higher frequency of severe disease and death, compared to those without hypertension. Similarly, patients with diabetes also had a higher frequency of severe disease and death compared to those without diabetes. Patients with other less prevalent comorbidities in this study which include the presence of existing kidney disease and heart disease also had a higher frequency of severe disease.

The comparison of baseline demographic and clinical parameters based on disease severity and outcome showed that patients with severe disease were older than those with the non-severe disease. It also showed that patients who died were also older. The impact of age on disease severity and mortality has been documented in prior studies [15,19]. These observations show the influences of age and comorbidities on severe disease and adverse outcomes as these patients are likely to have a combination of hypertension and diabetes, maybe older, and have other cardiovascular risk factors that increase the risk for critical care and poor outcomes. The patients with the severe disease also had higher values in baseline respiratory rate, baseline temperature, and baseline pulse rate. The finding of lower oxygen saturation (SPO_2_) in patients with severe disease and death was also reported by Shahriarirad *et al*. in Iran [9]. These observations indicate the utility of these factors in determining severe disease and predicting death.

## Conclusion

The clinical and epidemiologic characteristics of these hospitalised patients show significant similarities with existing trends from previously reported studies. This strengthens the evidence for a unified approach to infection prevention and case management. The spectrum of symptoms identified in this study parallels established patterns. However, the observation of hiccups and delirium shows the dynamic nature of COVID-19 symptomatology and calls for continued surveillance in monitoring symptoms of the disease. No age group is immune to COVID-19; prevention efforts are therefore applicable to all. However, older persons and those with comorbidities need infection prevention, early diagnosis, and aggressive treatment. It is anticipated that findings from this study will promote prevention, case investigation, and case management practices for COVID-19 in similar countries.

### What is known about this topic

- The clinical and epidemiologic patterns of COVID-19 which vary across geographic areas are influenced by socio-economic and demographic variables;
- The majority of patients have mild disease while leading symptoms are fever, cough, dyspnea, myalgia and anosmia;
- Age and comorbid disease conditions are associated with severe disease and adverse outcomes.

### What this study adds

- The epidemiologic and clinical characteristics of COVID-19 in Nigerians is similar to already established patterns in Africa and globally;
- The study shows that fever, dry cough, dyspnea, fatigue, productive cough, diarrhea, and vomiting are more associated with severe COVID-19 disease among Nigerians;
- This study shows that co-morbidities especially hypertension and diabetes and increasing age are associated with severe disease and death among Nigerians.
